# Recent Advances on the Roles of PCSK-9 Inhibitors in the Management of Acute Ischemic Stroke Patients

**DOI:** 10.3390/ijms231810221

**Published:** 2022-09-06

**Authors:** Silvina Ilut, Bianca O. Pirlog, Radu Pirlog, Andreea Nutu, Vitalie Vacaras, Sebastian M. Armean

**Affiliations:** 1 Department of Neuroscience, University of Medicine, and Pharmacy “Iuliu Hațieganu”, 400012 Cluj-Napoca, Romania; 2 Research Center for Functional Genomics, Biomedicine and Translational Medicine, University of Medicine, and Pharmacy “Iuliu Hațieganu”, 400337 Cluj-Napoca, Romania; 3 Department of Pharmacology, Toxicology and Clinical Pharmacology, University of Medicine, and Pharmacy “Iuliu Hațieganu”, 400337 Cluj-Napoca, Romania

**Keywords:** acute ischemic stroke, PCSK-9, statins, prevention, hypolipemic treatment, hyperlipemia

## Abstract

Acute ischemic stroke (AIS) represents an important cause of disability and death. Since only a minor percentage of patients with AIS are eligible for acute therapy, the management of risk factors is mandatory. An important risk factor of AIS is hyperlipemia. The current guidelines recommend a strict correction of it. Statins are recommended as the first-line treatment, while proprotein convertase subtilin/kexin type 9 (PCSK-9) inhibitors are administered as a second or even third option when the goal for a low-density lipoprotein cholesterol (LDL-C) level is not achieved. PCSK-9 inhibitors effectively decrease the LDL-C levels through the inhibition of PCSK-9-LDL-receptor complex formation. The in-depth understanding of the PCSK-9 protein mechanism in the metabolism of LDL-C led to the development of effective targeted approaches. Furthermore, a better understanding of the LDL-C metabolic pathway led to the development of newer approaches, which increased the therapeutic options. This article aims to offer an overview of the PCSK-9 inhibitors and their mechanism in reducing the LDL-C levels. Moreover, we will present the main indications of the current guidelines for patients with hyperlipemia and for those who have suffered an acute ischemic stroke, as well as the importance of LDL-C reduction in decreasing the rate of a recurrence.

## 1. Introduction

Acute ischemic stroke (AIS) is a leading cause of disability and death worldwide [1]. In Europe, approximately 1 million people experience an AIS every year [2]. The efficacy and safety of the acute therapeutic management (intravenous thrombolysis and endovascular treatment) is high; however, in Europe, only around 10% of AIS patients receive acute treatment [3]. In this context, an important role in the prevention of an AIS or recurrent AIS is the strict control of risk factors [4,5]. According to TOAST (Trial of Org 10172 in Acute Stroke Treatment) classification, the etiologies of AIS are the following: cardioembolic, large vessel atherosclerosis, small-vessel occlusion, other determined causes and cryptogenic [6]. In many situations the exact cause of AIS is hard to be determined, the reason for which the ASCOD (A—atherosclerosis, S—small-vessel disease; C—cardiac pathology; O—other causes, D—dissection) classification was introduced. This reinforced the importance of detecting and treating the risk factors that might favor AIS or AIS recurrence [7]. The well-known risk factors are hypertension, cardiac arrythmia, diabetes, obesity and hyperlipemia, and atherosclerosis, consequently [8]. However, the authors of the ASCOD classification suggest the goal of targeting LDL-C systematically in all types of AIS [7,9].

Hyperlipidemia is defined by an increased level (over 90th percentile) of the following parameters: total cholesterol, low-density lipoprotein cholesterol (LDL-C), triglyceride and a decreased level (less than 10th percentile) of high-density lipoprotein cholesterol (HDL-C) [10]. Multiple studies showed a direct relationship between the reduction in the LDL-C levels and the reduction in risk for atherosclerotic cardiovascular disease and cerebrovascular disease [11]. LDL-C became a treatment target more than twenty years ago [12]. LDL-C was found as a potential therapeutic target for hyperlipidemia in 1988 when the first Adult Treatment Panel I (ATP) guideline was published followed by ATP II, III and IV guidelines [13,14]. The initial goal for LDL-C in patients with atherosclerotic cardiovascular disease (ASCVD) was 130 mg/dL, while nowadays it is less than 55 mg/dL in the European guidelines [15] and less than 70 mg/dL in the North American guidelines [16].

Various therapeutic options are available nowadays proving the requested targets for LDL-C. The oldest drugs used for hyperlipidemia are statins, known as inhibitors of the hydroxymethyl-glutaryl-CoA (HMG-CoA) reductase enzyme [17], followed by ezetimibe, which inhibits the intestinal absorption of cholesterol, introduced as an add-on to the statin therapy [18]. Proprotein convertase subtilin/kexin type 9 (PCSK-9) inhibitors, are novel lipid-lowering drugs that have lately been taken more into consideration for patients not achieving the LDL-C threshold or who have statin intolerance [4,5,19]. PCSK-9 inhibitors function by binding to the PCSK-9 protein, therefore increasing the availability of LDL receptors (LDL-R) on the hepatocyte surface and lowering LDL-C levels [20,21]. Since their FDA approval in 2015, PCSK-9 inhibitors, have showed a significant reduction in LDL and a reduced risk of CV events [22,23,24]. A new class of PCSK-9 inhibitor is represented by inclisiran, a drug based on a silencing interfering RNA (siRNA), with a mechanism based on antisense nucleotides that target the PCSK-9 messenger RNA, inducing its degradation and a lower level of the PCSK-9 protein [25].

The current guidelines for hyperlipidemia recommend treatment initiation with statins, followed by a combination of statins and ezetimibe in the second line, and, if the aforementioned strategies fail, the introduction of PCSK-9 inhibitors [15,16].

The aim of this review is to provide an in-depth understanding of the action mechanisms, efficacy and safety of PCSK-9 inhibitors in targeting high LDL-C levels for the primary and secondary prevention of acute ischemic stroke, as well as to present the future directions regarding the use of this therapy for the management of stroke patients.

We conducted a literature review of the recently published literature from July 2015 since FDA approval to July 2022. The database search included PubMed and Cochrane using the following keywords: acute ischemic stroke, stroke, PCSK-9 inhibitors, secondary and primary prevention, alirocumab, evolocumab, bococizumab, inclisiran, hyperlipidemia. We included studies in English and on humans, mainly clinical trials, randomized controlled trials, systematic reviews and meta-analyses. References of the selected studies were searched manually for additional studies.

## 2. Mechanisms of the Hypolipidemic Treatments

**Figure 1:**
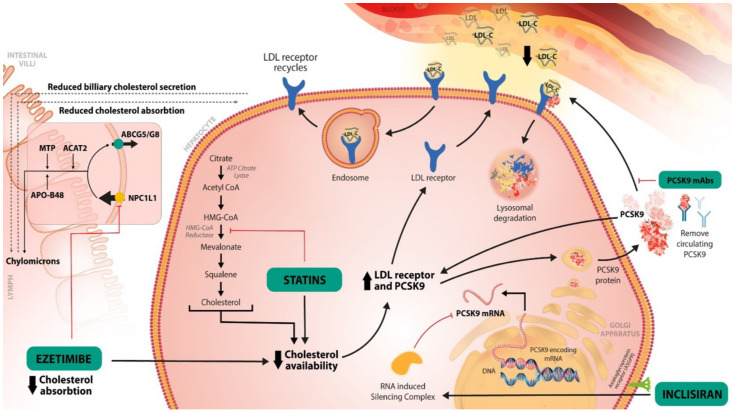
The main mechanisms of the LDL-C-lowering therapies. **Statins** directly inhibit the HMG-CoA reductase, thus inhibiting the transformation of HMG-CoA to mevalonate. Those are the precursors of cholesterols in its cascade of biosynthesis. By decreasing the cholesterol availability, more LDL-R will be recycled to the surface of hepatocyte, favoring the bind to LDL-C, thus reducing the LDL-C in the blood circulation. **Ezetimibe** inhibits the NPC1L1 protein, at the level of jejunal margin, thus the absorption of cholesterol will decrease. The cholesterol transported to the liver will also decrease. This will decrease the storage of cholesterol in the liver. Consequently, more LDL-R will be available to bind with LDL-C, decreasing again its level in the blood circulation. **PCSK-9 inhibitors** inhibit the binding of PCSK-9 protein to LDL-R. Consequently, the complex PCSK-9 protein-LDL-R will not form, and the LDL-R will not be degraded. Therefore, PCSK-9 inhibitors favor the recirculation of the LDL-R on the hepatocyte surface. **Inclisiran** prevents the formation of PCSK-9 protein, its mechanism is detailed in Figure 2.

Statins are a class of drugs that act as competitive inhibitors of the HMG-CoA reductase enzyme. This enzyme plays an important role in the initiation of the cholesterol synthetization cascade through the transformation of HMG-CoA into mevalonate [26]. Thus, the inhibition of HMG-CoA reductase with statins leads to a reduction in cholesterol synthesis in the liver and to an increase in LDL-R on the surface of a hepatocyte, favoring the uptake of the LDL-C from the blood circulation [27] (Figure 1). Statins are the first class recommended in the European Cardiology Society/European Atherosclerotic Society (ESC/EAS) and American College of Cardiology (ACC)/American Heart Association (AHA) guidelines for the therapeutic management of LDL-C levels [15,16]. Furthermore, the European and North American Stroke guidelines recommend statins as the first line of treatment for patients with hypercholesterolemia in both primary and secondary prevention [4,19].

Ezetimibe is a drug of the class of hypolipidemic drugs that act as selective inhibitors of cholesterol absorption at the intestinal level [28]. It targets the Niemann-Pick C1-Like 1 (NPC1L1) protein, which is localized at the apical membrane of enterocytes and at the canalicular membrane of hepatocytes; it mediates the intestinal cholesterol absorption and its hepato-biliary excretion [29]. Ezetimibe inhibits the NPC1L1 protein from the jejunal brush border, thus reducing the absorption of cholesterol into enterocytes. Consequently, the cholesterol from the liver is decreased, which favors an increase in the LDL-R on the surface of the hepatocyte, and a reduction in the LDL-C from the blood [18]. With a continuously changing goal for the LDL-C level in patients with hypercholesterolemia and ASCVD or AIS, a need for combinatoric therapy is required for patients that cannot reach the LDL-C goal with only high dose statins. The IMPROVE-IT trial showed the benefit of ezetimibe as an add-on to statin therapy in reaching an LDL-C lower than 70 mg/dL [30]. Additionally, the TST trial showed on a French cohort that patients with an LDL-C < 70 mg/dL had a lower risk of CV events [31]. The current guidelines for hypercholesterolemia and AIS secondary prevention recommend an association of statin to ezetimibe in patients with a very high risk of ASCVD if the LDL-C level is above 70 mg/dL with the maximum tolerated dose of statin [4,15,16,19]. If the LDL-C target cannot be reached with the statin–ezetimibe combination, the ESC/EAS and AHA guidelines indicate, as a 2A recommendation that is reasonable, to treat with PCSK-9 inhibitors to prevent further ASCVD events. They can be used as an add-on therapy or used in single therapy [4,15,16].

The PCSK-9 protein is synthetized in the liver and studies have shown that it plays an important role in the LDL-C metabolism through the inhibition of recirculation of the LDL-R [32]. The LDL-R are found at the surface of the hepatocyte and have the role of binding the LDL-C particles; the LDL-R-LDL-C complex is internalized into the cell and processed into an endosome. Next, the division of the two molecules occurs, with LDL-R being recycled to the surface of the cell, while the LDL-C particle enters the lysosomes for degradation [33]. When the PCSK-9 binds to LDL-R, the complex is internalized and directly degraded by the lysosome, resulting in a decrease in LDL-Rs at the hepatocyte surface, and thus, a decrease in LDL-C uptake [21]. The main effect of PCSK-9 inhibitors is to block the PCSK-9 proteins’ actions, enhancing the recycling of the LDL-R and the reduction in LDL-C, respectively [21,33,34]. PCSK-9 inhibitors have a potent effect on decreasing the LDL-C levels by approximately 60%; moreover, they have a synergic effect with other hypolipemic therapies (statins, ezetimibe) [34].

**Figure 2:**
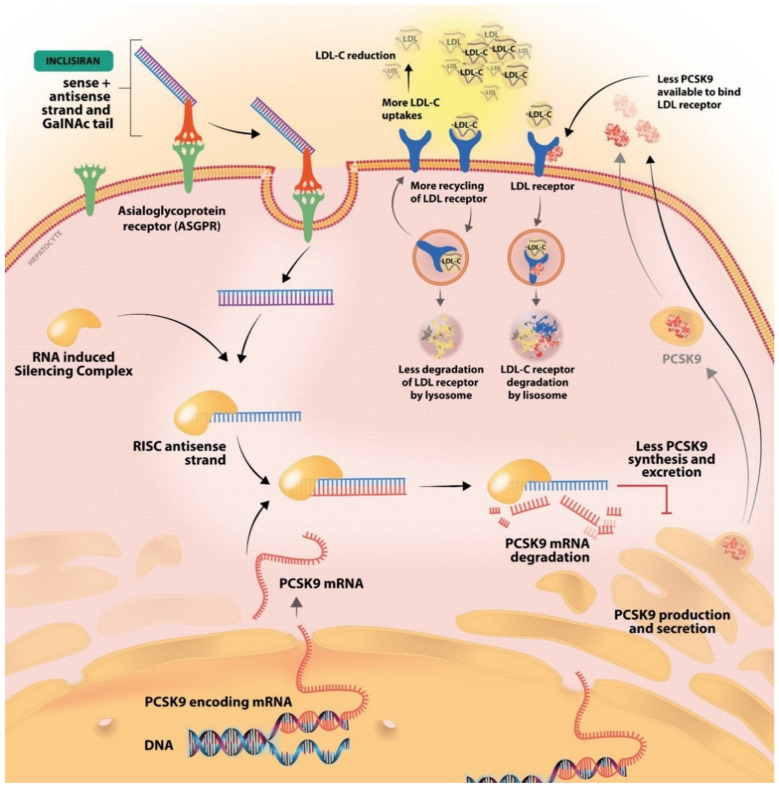
The mechanism of inclisiran, the first synthetic siRNA. After conjugation to GalNac, it binds the ASGPR receptor and is internalized into the cell. PCSK-9 mRNA is produced in the nucleus and then released into the cell. The internalized inclisiran binds the PCSK-9 mRNA, which leads to its degradation. Consequently, less PCSK-9 protein is synthesized and less will be available to bind the LDL-R. With an increased LDL-R available the LDL-C uptake will increase and the level of LDL-C in the blood circulation will decrease.

A novel PCSK-9 inhibitor is represented by inclisiran, which was approved by the EMA in 2020 and by the FDA in late 2021 [35,36]. Inclisiran targets the synthesis of the PCSK-9 protein using an siRNA approach [25]. siRNA are approximately 20–30 nucleotide RNA molecules that favor gene knockdown, thus decreasing the accumulation of the PSCK-9 protein by blocking the intracellular translation of PCSK-9 mRNA [37,38]. Inclisiran is a synthetic siRNA, with a long-acting effect, that is administered subcutaneously every 3 to 6 months [39]. The antisense oligonucleotides are conjugated to triantennary N-acetylgalactosamine carbohydrates (GalNac) that bind to liver-expressed asialoglycoprotein receptors, favoring the uptake of inclisiran into the cell [38,39,40]. Asialoglycoprotein receptor (ASGPR) is a transmembrane glycoprotein, mostly expressed in the liver, that mediates the uptake of compounds with N-acetylgalactosamine residues [41]. After internalization, inclisiran binds to the mRNA strand encoding for the PCSK-9 protein, creating a double strand RNA molecule that inhibits translation and induces its degradation (Figure 2) [42].

## 3. PCSK-9 Inhibitors in Clinical Practice

The main representative drugs of this class are evolocumab and alirocumab, which were approved by the FDA in 2015 as an add-on to the therapy for heterozygous familial hypercholesterolemia (HeFH) and homozygous familial hypercholesterolemia (HoFH) [43,44]. Bococizumab is another PCSK-9 inhibitor, but its production was discontinued due to its aberrant antidrug antibody production and limited effect on the reduction in LDL-C levels [45]. Inclisiran was the first siRNA treatment, approved by the EMA in 2020 and by FDA in 2021 to reduce LDL-C levels as an add-on therapy [35,46]. The advantages and disadvantages of different PCSK-9 inhibitors are presented in Table 1.

## 4. PCSK-9 Inhibitors in Clinical Trials

Alirocumab was the first FDA-approved PCSK-9 inhibitor drug [52]. The ODYSSEY OUTCOME study aimed to determine the efficacy and safety of alirocumab in the reduction in LDL-C levels and CV events. A regression of 59.5 to 62.0% for the LDL-C level compared to the placebo was registered and 79% of the enrolled patients achieved the LDL-C target of <70 mg/dL (as guideline recommended). Moreover, a reduction in major cardiovascular events (MACE) was observed. More neurocognitive events were registered in the alirocumab group, although without a statistical significance [23]. In an ODYSSEY OUTCOME sub-study designed to assess the effect of alirocumab in preventing an AIS, a net reduction in the risk of AIS occurrence was determined in the treatment group compared with placebo, and no hemorrhage was associated with it [53]. Furthermore, alirocumab had an increased effect on the non-fatal CV events and death at almost 3 of years follow-up [54]. 

Evolocumab was the next approved PCSK-9 inhibitor by the FDA in 2015 [47]. The FOURIER study included 27,564 patients with ASCVD with an LDL > 70 mg/dL or higher, who were receiving high or moderate doses of statin. Its main aim was to determine the efficacy and safety of evolocumab added to the statin therapy. There was an LDL-C reduction of 59% compared to the baseline values and a 15% reduction in the risk of the primary endpoint and a 20% reduction in the risk of AIS [22]. In a pre-specific sub-analysis of the FOURIER study on patients with a prior stroke, evolocumab as an add-on to the statin therapy significantly reduced the risk of recurrent AIS [55]. Moreover, the addition of evolocumab to standard therapy decreases the rate of acute arterial events in all territories [56]. In the GAUSS study, made on Japanese patients with hypercholesterolemia and intolerance to statins, evolocumab was superior to ezetimibe in decreasing LDL-C levels [57]. In the OSLER 1 study, patients who received evolocumab had a follow-up for 5 years showing their benefit and constant effect in LDL-C level reduction [58]. In a study that aimed to show the efficacy of evolocumab on coronary plaque, a reduction in the LDL-C level of 62.8% compared to a placebo was observed, and also a plaque reduction in the evolocumab group [59]. In a sub-analysis of the FOURIER study, the efficacy and safety of evolocumab seemed similar in both sexes at different ages [60]. As well as the net reduction in LDL-C level, evolocumab showed effects on the other lipid parameters as well [61,62].

After these two important clinical trials, various systematic reviews and meta-analyses were conducted to support the initial findings. In a meta-analysis based on 15 studies, PCSK-9 inhibitors reduced the levels of LDL-C between 54% and 74% compared to a placebo, respectively, and between 26% and 46% compared to ezetimibe. Moreover, evolocumab seemed to be superior to alirocumab in decreasing LDL-C levels with a reduction of almost 20% more [63]. Furthermore, evolocumab showed a similar beneficial effect between included patients of different races and ethnicities [64]. Several meta-analyses confirmed the net effect of PCSK-9 inhibitors in the significant reduction in LDL-C levels [65,66]. Moreover, the reduction in LDL-C levels with PCSK-9 therapy was associated with a decreased risk of both myocardial infarction (MI) and AIS and minimized the risk of recurrence [67]. In a Cochrane metanalysis, PSCK-9 inhibitors were found to reduce the risk of MI, AIS, and all-cause death compared to a placebo [68]. A pooled analysis that included both FOURIER and ODYSSEY OUTCOME studies, identified a reduced risk of stroke in patients with or without a previous stroke who received PCSK-9 inhibitors [69]. Similar results were obtained in several meta-analyses including patients who received PCSK-9 therapy [63,65,70,71,72]. Furthermore, patients with a low LDL-C level (<50 mg/dL) had the lowest risk of CV death, MI, or AIS [73].

The efficacy and safety of bococizumab, another PCSK-9 inhibitor, was tested in a clinical trial that included 27,438 patients, compared to a placebo. However, the study was terminated early by the sponsor. They observed a reduction in LDL-C level compared to the baseline by 64%, but there was no difference regarding the primary endpoint (non-fatal MI, non-fatal AIS, and death) compared to the placebo group. Moreover, more patients enrolled in the treatment group were discontinued due to adverse events [74]. Furthermore, it had a variable effect on LDL-C reduction, and half of the patients presented detectable antidrug antibodies at 1-year follow-up [75]. The drug production was discontinued at the end of 2016 [76].

Inclisiran is a novel drug in the PCSK-9 inhibitors’ class, based on antisense nucleotides conjugated with GalNac. Its safety and efficacy in lowering LDL-C levels were tested in a clinical trial that included 501 patients. Patients who received one dose had an LDL-C reduction of a maximum of 42%, while those who received two doses had a maximum reduction of 56% compared to baseline LDL-C. Moreover, it also played a role in the significant reduction in other cholesterol parameters [51]. Patients with HeFH who received inclisiran, also had an important LDL-C reduction, without any serious adverse events [77].

## 5. PCSK-9 Inhibitors and Adverse Reactions

As well as the above-mentioned common adverse events of the PCSK-9 inhibitors, two additional secondary side effects represent a concern in the use of PCSK-9 inhibitors. As already mentioned, PCSK-9 inhibitors induce low LDL-C and these side effects are assumed to be related to this, which is why some clinicians are more cautious with this therapy. These are represented by the neurocognitive adverse events and increased risk of hemorrhage, both supposedly associated with a low level of LDL-C. The concern regarding the association between low LDL-C and hemorrhage is questionable due to the results of multiple clinical trials that did not find an increased risk of intracerebral hemorrhage for patients with an LDL-C < 70 mg/dL [15,23,30,34,73]. In a metanalysis that pooled the data from the available randomized clinical trials, no significant difference was identified in terms of the risk of neurocognitive adverse effects and the risk of hemorrhage in patients receiving PCSK-9 inhibitors [72].

In two different leading studies regarding the efficacy and safety of PCSK-9 inhibitors, a slightly higher incidence of neurocognitive events was reported in the treatment groups. A direct correlation with the drug or indirect correlation with its effect on providing a low LDL-C level was suspected [23,48]. In a sub-analysis of the FOURIER STUDY, 1204 patients were included to be monitored for neurocognitive events. The analysis did not find a correlation between neurocognitive events and the usage of PCSK-9 inhibitors, nor in the patients with a very low LDL-C level [78]. Similar findings were found in another study that used a self-survey for detecting cognitive impairment, confirming the correlation between PCSK-9 treatment and neurocognitive events [79]. Another study that aimed to test the correlation between alirocumab and neurocognitive events included patients with HeFH or non-FH with very high CV risk and on treatment with a maximal dose of statin. Alirocumab did not show any correlation with neurocognitive events [80]. In a systematic review regarding lipid-lowering therapy and neurocognitive function, no differences were found between the group receiving PCSK-9 inhibitors and a placebo regarding the neurocognitive events [81]. In a meta-analysis that included 14 studies on alirocumab, the overall rate of neurocognitive events was low, similar to patients receiving a placebo [82]. These findings were also validated by multiple meta-analyses [65,70,71,83,84]. Furthermore, a low LDL-C level induced by PCSK-9 inhibitors did not increase the rate of cognitive performance [66]. On the other hand, one metanalysis pooled data from two outcomes studies (ODYSSEY LONG TERM and OSLER) and found a slight increase in the neurocognitive events in patients treated with PSCK-9 inhibitors [85].

A possible association between PCSK-9 inhibitors and glucose metabolism alteration has been suggested in the literature [86]. In patients with diabetes mellitus, an association between PCSK-9 levels and hemoglobin A1C levels was observed [87]. However, several studies on PCSK-9 inhibitors did not find any correlation between the usage of these drugs and the new onset of diabetes or hemoglobin A1C alteration [21,88,89]. Since the follow-up of these patients is relatively short in the above-mentioned studies, more research in this field is needed to exclude the suspected correlation.

## 6. Ongoing Clinical Trials Involving PCSK-9 Inhibitors and Future Directions

Several clinical trials with both evolocumab and alirocumab in patients with AIS are ongoing. Some are focusing on reducing the rate of stroke recurrence, while others aim to better understand either extra- or intracranial atherosclerotic plaque and its response to the CSK-9 mechanism. The ongoing clinical trials are presented in Table 2. The data regarding long follow-up for patients receiving this therapy in ASCVD or AIS are lacking and, unfortunately, at the moment of writing this article, no ongoing clinical trials are focusing on that. Moreover, there are no ongoing trials with inclisiran in AIS.

## 7. New Approaches for PCSK-9 Inhibition

Recently, the focus on molecular changes has increased. MicroRNAs (miRNA) are a class of small non-coding RNA of 18–28 nucleotides long that play an essential role in gene expression regulation. MiRNA dysregulation was found to have implications in various pathological conditions, including cancer, autoimmune disease, inflammatory disease, etc. [90,91]. Several miRNAs, such as miRNA-122, miRNA-185, and miRNA-182 seem to play a role in lipids metabolism by regulating the cholesterol synthesis in the hepatocyte and the LDL-R activity [92]. In a predictive study, miRNA-552-3p seems to be a potent PCSK-9 inhibitor both in vivo and in vitro, by direct targeting the PCSK-9 mRNA and protein, consequently [93]. Therefore, in the new clinical context of RNA-based therapeutics, it holds the promise of being investigated as a possible future siRNA-based therapy. 

More and more approaches are being developed for new classes of drugs that influence the reduction in LDL-C through the novel mechanism and are under evaluation due to the high cost of PCSK-9 inhibitors. CRISPR-based gene editing is a technology that can modulate PCSK-9 expression through genome editing [94,95]. Other therapeutic options are adnectins and anticalins that inhibit the interaction between the PCSK-9 protein and LDL-R [95,96,97]. Small molecules used as PCSK-9 inhibitors are meant to be a cheaper option than the PCSK-9 inhibitors, which will play a role in reducing the PCSK-9 protein secretion and interaction with LDL-R. There are several natural or synthetic small molecules such as berberine and guanidine derivates and amino-thiazole that seem to have a direct effect on the PCSK-9 mechanism [95,98]. Even though promising, these drugs require more research and clinical trials to prove their efficacy in clinical use.

## 8. Conclusions

Hyperlipidemia and atherosclerotic plaques represent an increased risk factor for AIS and its recurrence. However, various therapeutic options available nowadays are very efficacious in decreasing LDL-C levels and stabilizing atherosclerotic plaques. The PCSK-9 inhibitors favor the LDL-C reduction by more than 50%, and its effect is not influenced by sex or age [57,58,59,60,61,62,63,64,65,66,99]. As an add-on to standard therapy, they showed an important effect in reducing the incidence rate of AIS or its recurrence [48,53,67,73]. They also had an impact in decreasing the non-fatal CV and fatal events in a short-term follow-up (almost 3 years) [54]. The well-known syntagm “the lower the better” was proven in several studies, and it was found that patients with a lower level of LDL-C had fewer MI, AIS, and fatal events [73]. There seems to be a direct correlation between the low level of cholesterol and a reduction in AIS or AIS recurrence [34,100], justifying the indication for an LDL level < 55 mg/dL for this population [101]. Regarding the safety of PCSK-9 inhibitors, several studies and meta-analyses did not find an increased risk of hemorrhage correlated with a low level of LDL-C [15,23,34,73]. On the other hand, regarding the neurocognitive adverse effects, the data present discrepancies; however, in the pooled analysis, no correlation between the two was found [78,80,81,82,83,84,85].

In conclusion, PCSK-9 inhibitors play an important role in decreasing the rate of fatal and non-fatal CV events through the reduction in LDL-C levels, and their effect is even more potent than statins’ effect. Therefore, PCSK-9 inhibitors represent a viable option when the LDL-C goal is not achieved.

## Figures and Tables

**Table 1:** The representative drugs of the PCSK-9 inhibitors’ class and their characteristics.

Name	Indication	Administration	Benefit	Common Adverse Reactions	Ref.
Evolocumab	Primary and secondary hypercholesterolemia HoFH * Established ASCVD *	140 mg every 2 weeks subcutaneous or 420 mg once monthly	LDL-C level reduction by 61% Reduced CV events	Local injection site reaction Pruritus Upper respiratory tract symptoms	[47,48]
Alirocumab	Primary and secondary hypercholesterolemia Establish ASCVD *	75 mg every 2 weeks subcutaneous or 300 mg once monthly	LDL-C * level reduction by 62% decreased the rate of MACE *	Local injection site reaction Pruritus Upper respiratory tract symptoms	[23,49]
Inclisiran	Primary and secondary hypercholesterolemia	284 mg single administration subcutaneous, then repeated at 3 months, followed by every 6 months	LDL-C level reduction between 27% and 53%, depending on the number of doses	Local injection site reaction	[50,51]

* LDL-C = low-density lipoprotein cholesterol, ASCVD = atherosclerotic cardiovascular disease, MACE = major adverse cardiovascular events, HoFH = homozygous familial hypercholesterolemia.

**Table 2:** Ongoing trials on PCSK-9 inhibitors.

Nr. Crt.	Study Name	Number	Drug	Aim
1	EVOCAR-1 study	NCT03931161	Evolocumab	Determine the efficacy of evolocumab on carotid plaque morphology and composition in asymptomatic patients with >50% carotid artery stenosis.
2	CARUSO study	NCT04730973	Evolocumab	Determine the efficacy of evolocumab in promoting carotid plaque morphological stabilization and regression compared to traditional lipid-lowering therapy.
3	TOPICAL-MRI	NCT05001984	Alirocumab	Determine the efficacy and safety of alirocumab on patients with intracranial atherosclerotic plaque.
4	INSIST-HRMRI	NCT03753555	Evolocumab	Determine the effectiveness of intensive hypolipemic drug (statins vs. evolocumab, probucol) on patients with AIS and intracranial atherosclerotic plaque.
5	Study of Predictive Factors Related to Prognosis of Patients with Ischemic Stroke due to Large-artery Atherosclerosis	NCT04847752	PCSK-9 inhibitor	Determine the in-hospital factors that could predict the outcome of AIS patients and provide more evidence-based suggestions in the treatment and prognosis of atherosclerotic ischemic cerebrovascular disease.
6	sICASBLM	NCT05397405	PCSK-9 Inhibitors	Assess the impact of improving blood lipid management on clinical outcome of moderate to severe symptomatic intracranial atherosclerotic stenosis patients (LDL-C > 1.8 mmol/L) without endovascular therapy.

## References

[1] Tsao C.W., Aday A.W., Almarzooq Z.I., Alonso A., Beaton A.Z., Bittencourt M.S., Boehme A.K., Buxton A.E., Carson A.P., Commodore-Mensah Y. (2022). Heart Disease and Stroke Statistics—2022 Update: A Report From the American Heart Association. Circulation.

[2] Wafa H.A., Wolfe C.D., Emmett E., Roth G.A., Johnson C.O., Wang Y. (2020). Burden of Stroke in Europe. Stroke.

[3] De Sousa D.A., von Martial R., Abilleira S., Gattringer T., Kobayashi A., Gallofré M., Fazekas F., Szikora I., Feigin V., Caso V. (2018). Access to and delivery of acute ischaemic stroke treatments: A survey of national scientific societies and stroke experts in 44 European countries. Eur. Stroke J..

[4] Kleindorfer D.O., Towfighi A., Chaturvedi S., Cockroft K.M., Gutierrez J., Lombardi-Hill D., Kamel H., Kernan W.N., Kittner S.J., Leira E.C. (2021). 2021 Guideline for the Prevention of Stroke in Patients With Stroke and Transient Ischemic Attack: A Guideline From the American Heart Association/American Stroke Association. Stroke.

[5] Powers W.J., Rabinstein A.A., Ackerson T., Adeoye O.M., Bambakidis N.C., Becker K., Biller J., Brown M., Demaerschalk B.M., Hoh B. (2019). Guidelines for the Early Management of Patients With Acute Ischemic Stroke: 2019 Update to the 2018 Guidelines for the Early Management of Acute Ischemic Stroke: A Guideline for Healthcare Professionals From the American Heart Association/American Stroke Association. Stroke.

[6] Adams H.P., Bendixen B.H., Kappelle L.J., Biller J., Love B.B., Gordon D.L., Marsh E.E. (1993). Classification of subtype of acute ischemic stroke. Definitions for use in a multicenter clinical trial. TOAST. Trial of Org 10172 in Acute Stroke Treatment. Stroke.

[7] Sirimarco G., Lavallée P.C., Labreuche J., Meseguer E., Cabrejo L., Guidoux C., Klein I.F., Olivot J.-M., Abboud H., Adraï V. (2013). Overlap of Diseases Underlying Ischemic Stroke: The ASCOD Phenotyping. Stroke.

[8] Nelson R.H. (2012). Hyperlipidemia as a Risk Factor for Cardiovascular Disease. Prim. Care Clin. Off. Pract..

[9] Amarenco P., Bogousslavsky J., Caplan L., Donnan G., Wolf M., Hennerici M. (2013). The ASCOD Phenotyping of Ischemic Stroke (Updated ASCO Phenotyping). Cerebrovasc. Dis..

[10] Hill M.F., Bordoni B. (2022). Hyperlipidemia. StatPearls.

[11] Qin J., Liu L., Su X.D., Wang B.B., Fu B.S., Cui J.Z., Liu X.Y. (2021). The effect of PCSK9 inhibitors on brain stroke prevention: A systematic review and meta-analysis. Nutr. Metab. Cardiovasc. Dis..

[12] Safeer R.S., Ugalat P.S. (2002). Cholesterol treatment guidelines update. Am. Fam. Physician.

[13] Cleeman J.I., Grundy S.M. (1997). National Cholesterol Education Program Recommendations for Cholesterol Testing in Young Adults. Circulation.

[14] Grundy S.M., Cleeman J.I., Bairey Merz C.N., Brewer H.B., Clark L.T., Hunninghake D.B., Pasternak R.C., Smith S.C., Stone N.J. (2004). Implications of Recent Clinical Trials for the National Cholesterol Education Program Adult Treatment Panel III Guidelines. Circulation.

[15] Mach F., Baigent C., Catapano A.L., Koskinas K.C., Casula M., Badimon L., Chapman M.J., De Backer G.G., Delgado V., Ference B.A. (2019). 2019 ESC/EAS Guidelines for the management of dyslipidaemias: Lipid modification to reduce cardiovascular risk: The Task Force for the management of dyslipidaemias of the European Society of Cardiology (ESC) and European Atherosclerosis Society (EAS). Eur. Heart J..

[16] Grundy S.M., Stone N., Bailey A.L., Beam C., Birtcher K.K., Blumenthal R.S., Braun L.T., De Ferranti S., Faiella-Tommasino J., Forman D.E. (2019). 2018 AHA/ACC/AACVPR/AAPA/ABC/ACPM/ADA/AGS/APhA/ASPC/NLA/PCNA Guideline on the Management of Blood Cholesterol: A Report of the American College of Cardiology/American Heart Association Task Force on Clinical Practice Guidelines. Circulation.

[17] Sizar O., Khare S., Jamil R.T., Talati R. (2022). Statin Medications. StatPearls.

[18] Toth P., Phan B.A., Dayspring T. (2012). Ezetimibe therapy: Mechanism of action and clinical update. Vasc. Health Risk Manag..

[19] Dawson J., Béjot Y., Christensen L.M., De Marchis G.M., Dichgans M., Hagberg G., Heldner M.R., Milionis H., Li L., Pezzella F.R. (2022). European Stroke Organisation (ESO) guideline on pharmacological interventions for long-term secondary prevention after ischaemic stroke or transient ischaemic attack. Eur. Stroke J..

[20] Seidah N.G., Awan Z., Chrétien M., Mbikay M. (2014). PCSK9: A key modulator of cardiovascular health. Circ. Res..

[21] Handelsman Y., Lepor N.E. (2018). PCSK9 Inhibitors in Lipid Management of Patients With Diabetes Mellitus and High Cardiovascular Risk: A Review. J. Am. Heart Assoc..

[22] Sabatine M.S., Giugliano R.P., Keech A.C., Honarpour N., Wiviott S.D., Murphy S.A., Kuder J.F., Wang H., Liu T., Wasserman S.M. (2017). Evolocumab and Clinical Outcomes in Patients with Cardiovascular Disease. N. Engl. J. Med..

[23] Robinson J.G., Farnier M., Krempf M., Bergeron J., Luc G., Averna M., Stroes E.S., Langslet G., Raal F.J., El Shahawy M. (2015). Efficacy and Safety of Alirocumab in Reducing Lipids and Cardiovascular Events. N. Engl. J. Med..

[24] Board C., Kelly M., Shapiro M.D., Dixon D.L. (2020). PCSK9 Inhibitors in Secondary Prevention—An Opportunity for Personalized Therapy. J. Cardiovasc. Pharmacol..

[25] Dyrbuś K., Gąsior M., Penson P., Ray K.K., Banach M. (2020). Inclisiran—New hope in the management of lipid disorders?. J. Clin. Lipidol..

[26] Buhaescu I., Izzedine H. (2007). Mevalonate pathway: A review of clinical and therapeutical implications. Clin. Biochem..

[27] Ward N.C., Watts G.F., Eckel R.H. (2019). Statin Toxicity. Mechanistic Insights and Clinical Implications. Circ. Res..

[28] Nutescu E.A., Shapiro N.L. (2003). Ezetimibe: A Selective Cholesterol Absorption Inhibitor. Pharmacother. J. Hum. Pharmacol. Drug Ther..

[29] Jia L., Betters J.L., Yu L. (2011). Niemann-Pick C1-Like 1 (NPC1L1) Protein in Intestinal and Hepatic Cholesterol Transport. Annu. Rev. Physiol..

[30] Cannon C.P., Blazing M.A., Giugliano R.P., McCagg A., White J.A., Théroux P., Darius H., Lewis B.S., Ophuis T.O., Jukema J.W. (2015). Ezetimibe Added to Statin Therapy after Acute Coronary Syndromes. N. Engl. J. Med..

[31] Amarenco P., Kim J.S., Labreuche J., Charles H., Abtan J., Béjot Y., Cabrejo L., Cha J.-K., Ducrocq G., Giroud M. (2020). A Comparison of Two LDL Cholesterol Targets after Ischemic Stroke. N. Engl. J. Med..

[32] Cariou B., Le May C., Costet P. (2011). Clinical aspects of PCSK9. Atherosclerosis.

[33] Roth E.M., Davidson M.H. (2018). PCSK9 Inhibitors: Mechanism of Action, Efficacy, and Safety. Rev. Cardiovasc. Med..

[34] Gil-Núñez A., Masjuan J., Montaner J., Castellanos M., Segura T., Cardona P., Tembl J., Purroy F., Arenillas J., Palacio E. (2021). Proprotein convertase subtilisin/kexin type 9 inhibitors in secondary prevention of vascular events in patients with stroke: Consensus document and practice guidance. Neurologia.

[35] Lamb Y.N. (2021). Inclisiran: First Approval. Drugs.

[36] FDA Update: Inclisiran Approved as Add-On Therapy to Reduce LDL-C in High-Risk Adults. https://www.acc.org/Latest-in-Cardiology/Articles/2022/01/12/15/21/http%3a%2f%2fwww.acc.org%2fLatest-in-Cardiology%2fArticles%2f2022%2f01%2f12%2f15%2f21%2fFDA-Update-Inclisiran-Approved-as-Add-On-Therapy-to-Reduce-LDL-C-in-High-Risk-Adults.

[37] Alshaer W., Zureigat H., Al Karaki A., Al-Kadash A., Gharaibeh L., Hatmal M.M., Aljabali A.A., Awidi A. (2021). siRNA: Mechanism of action, challenges, and therapeutic approaches. Eur. J. Pharmacol..

[38] German C.A., Shapiro M.D. (2019). Small Interfering RNA Therapeutic Inclisiran: A New Approach to Targeting PCSK9. BioDrugs Clin. Immunother. Biopharm. Gene Ther..

[39] Fitzgerald K., White S., Borodovsky A., Bettencourt B.R., Strahs A., Clausen V., Wijngaard P., Horton J.D., Taubel J., Brooks A. (2017). A Highly Durable RNAi Therapeutic Inhibitor of PCSK9. N. Engl. J. Med..

[40] Nair J.K., Willoughby J.L.S., Chan A., Charisse K., Alam R., Wang Q., Hoekstra M., Kandasamy P., Kel’In A.V., Milstein S. (2014). Multivalent *N*-Acetylgalactosamine-Conjugated siRNA Localizes in Hepatocytes and Elicits Robust RNAi-Mediated Gene Silencing. J. Am. Chem. Soc..

[41] Susan-Resiga D., Girard E., Essalmani R., Roubtsova A., Marcinkiewicz J., Derbali R.M., Evagelidis A., Byun J.H., Lebeau P.F., Austin R.C. (2021). Asialoglycoprotein receptor 1 is a novel PCSK9-independent ligand of liver LDLR cleaved by furin. J. Biol. Chem..

[42] Henney N.C., Banach M., Penson P.E. (2021). RNA Silencing in the Management of Dyslipidemias. Curr. Atheroscler. Rep..

[43] U.S. Food and Drug Administration (2021). FDA Approves Add-On Drug for Ages 10 & Up with Rare Forms of High Cholesterol.

[44] U.S. Food and Drug Administration (2021). FDA Approves Add-On Therapy for Patients with Genetic Form of Severely High Cholesterol.

[45] Ferri N., Corsini A., Sirtori C.R., Ruscica M. (2016). Bococizumab for the treatment of hypercholesterolaemia. Expert Opin. Biol. Ther..

[46] U.S. Food and Drug Administration (2021). FDA Approves Add-On Therapy to Lower Cholesterol among Certain High-Risk Adults.

[47] EMA Repatha. https://www.ema.europa.eu/en/medicines/human/EPAR/repatha.

[48] Sabatine M.S., Giugliano R.P., Wiviott S.D., Raal F.J., Blom D.J., Robinson J., Ballantyne C.M., Somaratne R., Legg J., Wasserman S.M. (2015). Efficacy and Safety of Evolocumab in Reducing Lipids and Cardiovascular Events. N. Engl. J. Med..

[49] EMA Praluent. https://www.ema.europa.eu/en/medicines/human/EPAR/praluent.

[50] EMA Leqvio. https://www.ema.europa.eu/en/medicines/human/EPAR/leqvio.

[51] Ray K.K., Landmesser U., Leiter L.A., Kallend D., Dufour R., Karakas M., Hall T., Troquay R.P., Turner T., Visseren F.L. (2017). Inclisiran in Patients at High Cardiovascular Risk with Elevated LDL Cholesterol. N. Engl. J. Med..

[52] Raedler L.A. (2016). Praluent (Alirocumab): First PCSK9 Inhibitor Approved by the FDA for Hypercholesterolemia. Am. Health Drug Benefits.

[53] Jukema J.W., Zijlstra L.E., Bhatt D.L., Bittner V.A., Diaz R., Drexel H., Goodman S.G., Kim Y.-U., Pordy R., Reiner Ž. (2019). Effect of Alirocumab on Stroke in ODYSSEY OUTCOMES. Circulation.

[54] Szarek M., White H.D., Schwartz G.G., Alings M., Bhatt D.L., Bittner V.A., Chiang C.-E., Diaz R., Edelberg J.M., Goodman S.G. (2018). Alirocumab Reduces Total Nonfatal Cardiovascular and Fatal Events: The ODYSSEY OUTCOMES Trial. J. Am. Coll. Cardiol..

[55] Giugliano R.P., Pedersen T.R., Saver J.L., Sever P.S., Keech A.C., Bohula E.A., Murphy S.A., Wasserman S.M., Honarpour N., Wang H. (2020). Stroke Prevention With the PCSK9 (Proprotein Convertase Subtilisin-Kexin Type 9) Inhibitor Evolocumab Added to Statin in High-Risk Patients With Stable Atherosclerosis. Stroke.

[56] Oyama K., Giugliano R.P., Tang M., Bonaca M.P., Saver J.L., Murphy S.A., Ruzza A., Keech A.C., Sever P.S., Sabatine M.S. (2021). Effect of evolocumab on acute arterial events across all vascular territories: Results from the FOURIER trial. Eur. Heart J..

[57] Koba S., Inoue I., Cyrille M., Lu C., Inomata H., Shimauchi J., Kajinami K. (2020). Evolocumab vs. Ezetimibe in Statin-Intolerant Hyperlipidemic Japanese Patients: Phase 3 GAUSS-4 Trial. J. Atheroscler. Thromb..

[58] Koren M.J., Sabatine M.S., Giugliano R.P., Langslet G., Wiviott S.D., Ruzza A., Ma Y., Hamer A.W., Wasserman S.M., Raal F.J. (2019). Long-Term Efficacy and Safety of Evolocumab in Patients With Hypercholesterolemia. J. Am. Coll. Cardiol..

[59] Nicholls S.J., Puri R., Anderson T., Ballantyne C.M., Cho L., Kastelein J.J., Koenig W., Somaratne R., Kassahun H., Yang J. (2018). Effect of Evolocumab on Coronary Plaque Composition. J. Am. Coll. Cardiol..

[60] Sever P., Gouni-Berthold I., Keech A., Giugliano R., Pedersen T.R., Im K., Wang H., Knusel B., Sabatine M.S., O’Donoghue M.L. (2020). LDL-cholesterol lowering with evolocumab, and outcomes according to age and sex in patients in the FOURIER Trial. Eur. J. Prev. Cardiol..

[61] Alhajri L., Alhadhrami A., Almheiri S., Almutawa Y., Alhashimi Z. (2017). The efficacy of evolocumab in the management of hyperlipidemia: A systematic review. Ther. Adv. Cardiovasc. Dis..

[62] Toth P.P., Sattar N., Blom D.J., Martin S.S., Jones S.R., Monsalvo M.L., Elliott M., Davis M., Somaratne R., Preiss D. (2017). Effect of Evolocumab on Lipoprotein Particles. Am. J. Cardiol..

[63] Toth P.P., Worthy G., Gandra S.R., Sattar N., Bray S., Cheng L., Bridges I., Worth G.M., Dent R., Forbes C.A. (2017). Systematic Review and Network Meta-Analysis on the Efficacy of Evolocumab and Other Therapies for the Management of Lipid Levels in Hyperlipidemia. J. Am. Heart Assoc..

[64] Daviglus M.L., Ferdinand K.C., López J.A.G., Wu Y., Monsalvo M.L., Rodriguez C.J. (2021). Effects of Evolocumab on Low-Density Lipoprotein Cholesterol, Non–High Density Lipoprotein Cholesterol, Apolipoprotein B, and Lipoprotein(a) by Race and Ethnicity: A Meta-Analysis of Individual Participant Data From Double-Blind and Open-Label Extension Studies. J. Am. Heart Assoc..

[65] Talasaz A.H., Ho A.C., Bhatty F., Koenig R.A., Dixon D.L., Baker W.L., Van Tassell B.W. (2021). Meta-analysis of clinical outcomes of PCSK9 modulators in patients with established ASCVD. Pharmacother. J. Hum. Pharmacol. Drug Ther..

[66] Ying H., Wang J., Shen Z., Wang M., Zhou B. (2020). Impact of Lowering Low-Density Lipoprotein Cholesterol with Contemporary Lipid-Lowering Medicines on Cognitive Function: A Systematic Review and Meta-Analysis. Cardiovasc. Drugs Ther..

[67] Murphy S.A., Pedersen T.R., Gaciong Z.A., Ceska R., Ezhov M.V., Connolly D.L., Jukema J.W., Toth K., Tikkanen M.J., Im K. (2019). Effect of the PCSK9 Inhibitor Evolocumab on Total Cardiovascular Events in Patients With Cardiovascular Disease. JAMA Cardiol..

[68] Schmidt A.F., Carter J.-P.L., Pearce L.S., Wilkins J.T., Overington J.P., Hingorani A.D., Casas J. (2020). PCSK9 monoclonal antibodies for the primary and secondary prevention of cardiovascular disease. Cochrane Database Syst. Rev..

[69] Sagris D., Ntaios G., Georgiopoulos G., Pateras K., Milionis H. (2021). Proprotein Convertase Subtilisin-Kexin Type 9 inhibitors and stroke prevention: A meta-analysis. Eur. J. Intern. Med..

[70] Bajaj N.S., Patel N., Kalra R., Ahmad A., Venkatraman A., Arora G., Arora P. (2017). Neurological effects of proprotein convertase subtilisin/kexin type 9 inhibitors: Direct comparisons. Eur. Heart J. Qual. Care Clin. Outcomes.

[71] Karatasakis A., Danek B.A., Karacsonyi J., Rangan B.V., Roesle M.K., Knickelbine T., Miedema M.D., Khalili H., Ahmad Z., Abdullah S. (2017). Effect of PCSK9 Inhibitors on Clinical Outcomes in Patients With Hypercholesterolemia: A Meta-Analysis of 35 Randomized Controlled Trials. J. Am. Heart Assoc..

[72] Guedeney P., Giustino G., Sorrentino S., Claessen B.E., Camaj A., Kalkman D.N., Vogel B., Sartori S., De Rosa S., Baber U. (2019). Efficacy and safety of alirocumab and evolocumab: A systematic review and meta-analysis of randomized controlled trials. Eur. Heart J..

[73] Giugliano R.P., Pedersen T.R., Park J.-G., De Ferrari G.M., Gaciong Z.A., Ceska R., Toth K., Gouni-Berthold I., Lopez-Miranda J., Schiele F. (2017). Clinical efficacy and safety of achieving very low LDL-cholesterol concentrations with the PCSK9 inhibitor evolocumab: A prespecified secondary analysis of the FOURIER trial. Lancet.

[74] Ridker P.M., Revkin J., Amarenco P., Brunell R., Curto M., Civeira F., Flather M., Glynn R.J., Gregoire J., Jukema J.W. (2017). Cardiovascular Efficacy and Safety of Bococizumab in High-Risk Patients. N. Engl. J. Med..

[75] Ridker P.M., Tardif J.-C., Amarenco P., Duggan W., Glynn R.J., Jukema J.W., Kastelein J.J., Kim A.M., Koenig W., Nissen S. (2017). Lipid-Reduction Variability and Antidrug-Antibody Formation with Bococizumab. N. Engl. J. Med..

[76] Pfizer Discontinues Global Development of Bococizumab, Its Investigational PCSK9 Inhibitor | Pfizer. https://www.pfizer.com/news/press-release/press-release-detail/pfizer_discontinues_global_development_of_bococizumab_its_investigational_pcsk9_inhibitor.

[77] Raal F.J., Kallend D., Ray K.K., Turner T., Koenig W., Wright R.S., Wijngaard P.L., Curcio D., Jaros M.J., Leiter L.A. (2020). Inclisiran for the Treatment of Heterozygous Familial Hypercholesterolemia. N. Engl. J. Med..

[78] Giugliano R.P., Mach F., Zavitz K., Kurtz C., Im K., Kanevsky E., Schneider J., Wang H., Keech A., Pedersen T.R. (2017). Cognitive Function in a Randomized Trial of Evolocumab. N. Engl. J. Med..

[79] Gencer B., Mach F., Guo J., Im K., Ruzza A., Wang H., Kurtz C.E., Pedersen T.R., Keech A.C., Ott B.R. (2020). Cognition After Lowering LDL-Cholesterol With Evolocumab. J. Am. Coll. Cardiol..

[80] Janik M.J., Urbach D.V., van Nieuwenhuizen E., Zhao J., Yellin O., Baccara-Dinet M.T., Pordy R., Manvelian G. (2021). Alirocumab treatment and neurocognitive function according to the CANTAB scale in patients at increased cardiovascular risk: A prospective, randomized, placebo-controlled study. Atherosclerosis.

[81] Kyriakos G., Quiles-Sánchez L.V., Diamantis E., Farmaki P., Garmpis N., Damaskos C., Savvanis S., Patsouras A., Stelianidi A., Voutyritsa E. (2020). Lipid-lowering Drugs and Neurocognitive Function: A Systematic Review. In Vivo.

[82] Harvey P.D., Sabbagh M.N., Harrison J.E., Ginsberg H.N., Chapman M.J., Manvelian G., Moryusef A., Mandel J., Farnier M. (2017). No evidence of neurocognitive adverse events associated with alirocumab treatment in 3340 patients from 14 randomized Phase 2 and 3 controlled trials: A meta-analysis of individual patient data. Eur. Heart J..

[83] Raccah B.H., Yanovsky A., Treves N., Rotshild V., Renoux C., Danenberg H., Eliaz R., Matok I. (2021). Proprotein Convertase Subtilisin/Kexin Type 9 (PCSK9) inhibitors and the risk for neurocognitive adverse events: A systematic review, meta-analysis and meta-regression. Int. J. Cardiol..

[84] Van Bruggen F.H., Nijhuis G.B.J., Zuidema S.U., Luijendijk H. (2020). Serious adverse events and deaths in PCSK9 inhibitor trials reported on ClinicalTrials.gov: A systematic review. Expert Rev. Clin. Pharmacol..

[85] Khan A.R., Bavishi C., Riaz H., Farid T.A., Khan S., Atlas M., Hirsch G., Ikram S., Bolli R. (2017). Increased Risk of Adverse Neurocognitive Outcomes With Proprotein Convertase Subtilisin-Kexin Type 9 Inhibitors. Circ. Cardiovasc. Qual. Outcomes.

[86] Da Dalt L., Ruscica M., Bonacina F., Balzarotti G., Dhyani A., Di Cairano E., Baragetti A., Arnaboldi L., De Metrio S., Pellegatta F. (2019). PCSK9 deficiency reduces insulin secretion and promotes glucose intolerance: The role of the low-density lipoprotein receptor. Eur. Heart J..

[87] Yang S.-H., Li S., Zhang Y., Xu R.-X., Guo Y.-L., Zhu C.-G., Wu N.-Q., Cui C.-J., Sun J., Li J.-J. (2015). Positive correlation of plasma PCSK9 levels with HbA_1c_ in patients with type 2 diabetes. Diabetes/Metab. Res. Rev..

[88] Nicholls S.J., Puri R., Anderson T., Ballantyne C.M., Cho L., Kastelein J.J.P., Koenig W., Somaratne R., Kassahun H., Yang J. (2016). Effect of Evolocumab on Progression of Coronary Disease in Statin-Treated Patients: The GLAGOV Randomized Clinical Trial. JAMA.

[89] Deedwania P., Murphy S.A., Scheen A., Badariene J., Pineda A.L., Honarpour N., Keech A.C., Sever P.S., Pedersen T.R., Sabatine M.S. (2021). Efficacy and Safety of PCSK9 Inhibition With Evolocumab in Reducing Cardiovascular Events in Patients With Metabolic Syndrome Receiving Statin Therapy: Secondary Analysis From the FOURIER Randomized Clinical Trial. JAMA Cardiol..

[90] O’Brien J., Hayder H., Zayed Y., Peng C. (2018). Overview of MicroRNA Biogenesis, Mechanisms of Actions, and Circulation. Front. Endocrinol..

[91] Ardekani A.M., Naeini M.M. (2010). The Role of MicroRNAs in Human Diseases. Avicenna J. Med. Biotechnol..

[92] Goedeke L., Aranda J.F., Fernández-Hernando C. (2014). microRNA regulation of lipoprotein metabolism. Curr. Opin. Lipidol..

[93] Ma N., Fan L., Dong Y., Xu X., Yu C., Chen J., Ren J. (2021). New PCSK9 inhibitor miR-552-3p reduces LDL-C via enhancing LDLR in high fat diet-fed mice. Pharmacol. Res..

[94] Pal M., Herold M.J. (2021). CRISPR base editing applications for identifying cancer-driving mutations. Biochem. Soc. Trans..

[95] Ahamad S., Mathew S., Khan W.A., Mohanan K. (2022). Development of small-molecule PCSK9 inhibitors for the treatment of hypercholesterolemia. Drug Discov. Today.

[96] Masuda Y., Yamaguchi S., Suzuki C., Aburatani T., Nagano Y., Miyauchi R., Suzuki E., Yamamura N., Nagatomo K., Ishihara H. (2018). Generation and Characterization of a Novel Small Biologic Alternative to Proprotein Convertase Subtilisin/Kexin Type 9 (PCSK9) Antibodies, DS-9001a, Albumin Binding Domain–Fused Anticalin Protein. J. Pharmacol. Exp. Ther..

[97] Mitchell T., Chao G., Sitkoff D., Lo F., Monshizadegan H., Meyers D., Low S., Russo K., DiBella R., Denhez F. (2014). Pharmacologic Profile of the Adnectin BMS-962476, a Small Protein Biologic Alternative to PCSK9 Antibodies for Low-Density Lipoprotein Lowering. J. Pharmacol. Exp. Ther..

[98] Abdul-Rahman T., Bukhari S.M.A., Herrera E.C., Awuah W.A., Lawrence J., de Andrade H., Patel N., Shah R., Shaikh R., Capriles C.A.A. (2022). Lipid Lowering Therapy: An Era Beyond Statins. Curr. Probl. Cardiol..

[99] Blom D.J., Hala T., Bolognese M., Lillestol M.J., Toth P.D., Burgess L., Ceska R., Roth E., Koren M.J., Ballantyne C.M. (2014). A 52-Week Placebo-Controlled Trial of Evolocumab in Hyperlipidemia. N. Engl. J. Med..

[100] Salvatore T., Morganti R., Marchioli R., De Caterina R. (2019). Cholesterol Lowering and Stroke: No Longer Room for Pleiotropic Effects of Statins—Confirmation from PCSK9 Inhibitor Studies. Am. J. Med..

[101] Tsankof A., Tziomalos K. (2021). The Role of Lipid-Lowering Treatment in the Secondary Prevention of Ischemic Stroke. Diseases.

